# Resveratrol Attenuates Early Brain Injury after Experimental Subarachnoid Hemorrhage via Inhibition of NLRP3 Inflammasome Activation

**DOI:** 10.3389/fnins.2017.00611

**Published:** 2017-11-03

**Authors:** Xiangsheng Zhang, Qi Wu, Qingrong Zhang, Yue Lu, Jingpeng Liu, Wei Li, Shengyin Lv, Mengliang Zhou, Xin Zhang, Chunhua Hang

**Affiliations:** ^1^Department of Neurosurgery, Jinling Hospital, School of Medicine, Nanjing University, Nanjing, China; ^2^Department of Neurosurgery, Jinling Hospital, School of Medicine, Southern Medical University, Nanjing, China

**Keywords:** subarachnoid hemorrhage, early brain injury, resveratrol, inflammation, NLRP3

## Abstract

Previous studies have demonstrated resveratrol (RSV) has beneficial effects in early brain injury (EBI) after subarachnoid hemorrhage (SAH). However, the beneficial effects of RSV and the underlying mechanisms have not been clearly identified. The nucleotide-binding oligomerization domain-like receptor family pyrin domain-containing 3 (NLRP3) inflammasome activation plays a crucial role in the EBI pathogenesis. The aim of this study was to investigate the role of RSV on the NLRP3 inflammasome signaling pathway and EBI in rats after SAH. A prechiasmatic cistern injection model was established in rats, and the primary cultured cortical neurons were stimulated with oxyhemoglobin (oxyHb) to induce SAH *in vitro*. It showed that the NLRP3 inflammasome components, including NLRP3, apoptosis-associated speck-like protein containing a caspase recruitment domain (ASC), caspase-1, mature interleukin-1β (IL-1β), and interleukin-18 (IL-18) were upregulated after SAH, and the enhanced NLRP3 after SAH was mainly located in microglia. Treatment with 60 or 90 mg/kg RSV after SAH dramatically inhibited the expression of NLRP3, but there was no significant difference in the expression of NLRP3 between the SAH + 60 mg/kg RSV and SAH + 90 mg/kg RSV groups. In addition, treatment with 30 mg/kg RSV did not significantly reduced the expression of NLRP3. We next evaluated the neuroprotective effects of RSV against SAH. We determined that SAH-induced NLRP3 inflammasome activation was significantly inhibited in the SAH + 60 mg/kg RSV group. Meanwhile, 60 mg/kg RSV administration could markedly inhibit microglia activation and neutrophils infiltration after SAH. Concomitant with the decreased cerebral inflammation, RSV evidently reduced cortical apoptosis, brain edema, and neurobehavioral impairment after SAH. *In vitro* experiments, RSV treatment also clearly protected primary cortical neurons against oxyHb insults, including reduced the proportion of neuronal apoptosis, alleviated neuronal degeneration, and improved cell viabilities. These *in vitro* data further confirm that RSV has an efficient neuroprotection against SAH. Taken together, these *in vivo* and *in vitro* findings suggested RSV could protect against EBI after SAH, at least partially via inhibiting NLRP3 inflammasome signaling pathway.

## Introduction

Subarachnoid hemorrhage (SAH) is a life-threatening disease and accounts for about 6–8% of all human strokes (Sehba et al., 2011; Li J. et al., 2015). Currently, early brain injury (EBI) is one of the key mechanisms of SAH and plays a critical role in high mortality and disability after SAH (Sehba et al., 2011; Chen et al., 2014; Yuan et al., 2017). A growing body of evidence indicates that inflammatory injury plays pivotal roles in the EBI pathogenesis and may represent for a promising goal for therapeutic intervention following SAH (You et al., 2016; Zhang et al., 2016b; Wu Q. et al., 2017).

Resveratrol (RSV), a natural occurring polyphenolic compound extracted from pines and grapevines, can easily pass the blood-brain barrier (Zhang et al., 2016a). Its wide range of biological properties, such as anti-inflammatory, anti-oxidant, and anti-tumor activity, has been extensively studied in different research fields (Fu et al., 2013; Shao et al., 2014; Zhang T. et al., 2014). In the central nervous system (CNS) diseases including traumatic brain injury (TBI), cerebral ischemia and neurodegenerative diseases, RSV has been shown to have promising neuroprotective effects due to its multiple functions (Della-Morte et al., 2009; Lin et al., 2014; Rege et al., 2014). Furthermore, recent researches have also demonstrated that RSV protects against EBI after SAH due to its powerful anti-inflammatory property (Shao et al., 2014; Zhang et al., 2016a). However, the anti-inflammatory effects of RSV and the potential molecular mechanisms have not been fully investigated in EBI after SAH.

The nucleotide-binding oligomerization domain-like receptor (NLR) family pyrin domain-containing 3 (NLRP3) inflammasome, a core component of the innate immune system, can facilitate caspase-1, interleukin-1β (IL-1β) and interleukin-18 (IL-18) processing, and then amplify the inflammatory response (Chen et al., 2013). Recently, mounting evidence has demonstrated NLRP3 inflammasome plays a key role in a variety of inflammatory diseases, such as atherosclerosis, metabolic syndrome, cardiovascular disorders, and stroke (Chen et al., 2013; Coll et al., 2015). In SAH, NLRP3 inflammasome activation is also essential for the modulation of pro-inflammatory cytokines, and inhibition NLRP3 inflammasome by pharmacological treatment could protect against brain injury after SAH (Chen et al., 2013; Li J. et al., 2015; Dong et al., 2016). In addition, the activation of NLRP3 inflammasome is associated with the frequency of apoptosis, and inhibition NLRP3 inflammasome could suppress cell apoptosis (Wali et al., 2013; Wu et al., 2014; Lebeaupin et al., 2015; Li Y. et al., 2015; Dong et al., 2016). However, until now, no study has been performed to investigate whether RSV could influence NLRP3 inflammasome-related inflammatory pathway during SAH. Thus, the aim of the present study was to explore the role of NLRP3 inflammasome in the therapeutic effects of RSV in EBI after SAH.

## Materials and methods

### Animals

Adult male Sprague-Dawley rats weighing 250–300 g and 15–18-day-old pregnant C57BL/6 mice were purchased from the Animal Center of Jinling Hospital (Nanjing, China). All experimental protocols were approved by the Animal Care and Use Committee of Nanjing University and in accordance with the Guide for the Care and Use of Laboratory Animals by National Institutes of Health (Wu L. Y. et al., 2017).

### Primary neuron culture

Primary cortical neurons were cultured as described previously (Sun et al., 2014; Wang Z. et al., 2016). Briefly, cerebral cortex was isolated from brains of fetal mice. The blood vessels and meninges were removed and brain tissues were digested with 0.25% trypsin for 5 min at 37°C. Then, the supernatant containing trypsin was discarded and washed with pre-cooling phosphate buffered saline (PBS). After that, brain tissue suspensions were passed through a 22 μm-filter and centrifuged at 1,500 r/min for 5 min. Subsequently, the cells were distributed in poly-D-lysine-coated plates and suspended in Neurobasal media supplemented with B27, glutamate, Hepes, penicillin and streptomycin (all bought from Gibco Incorporation). Cultures were maintained in a humidified incubator with 5% CO_2_ and 95% air at 37°C. Finally, half of the medium was replaced with fresh medium every 2 days. The *in vitro* studies were performed with neurons that had been in culture for 8–10 days.

### SAH rat model

**Figure 1:**
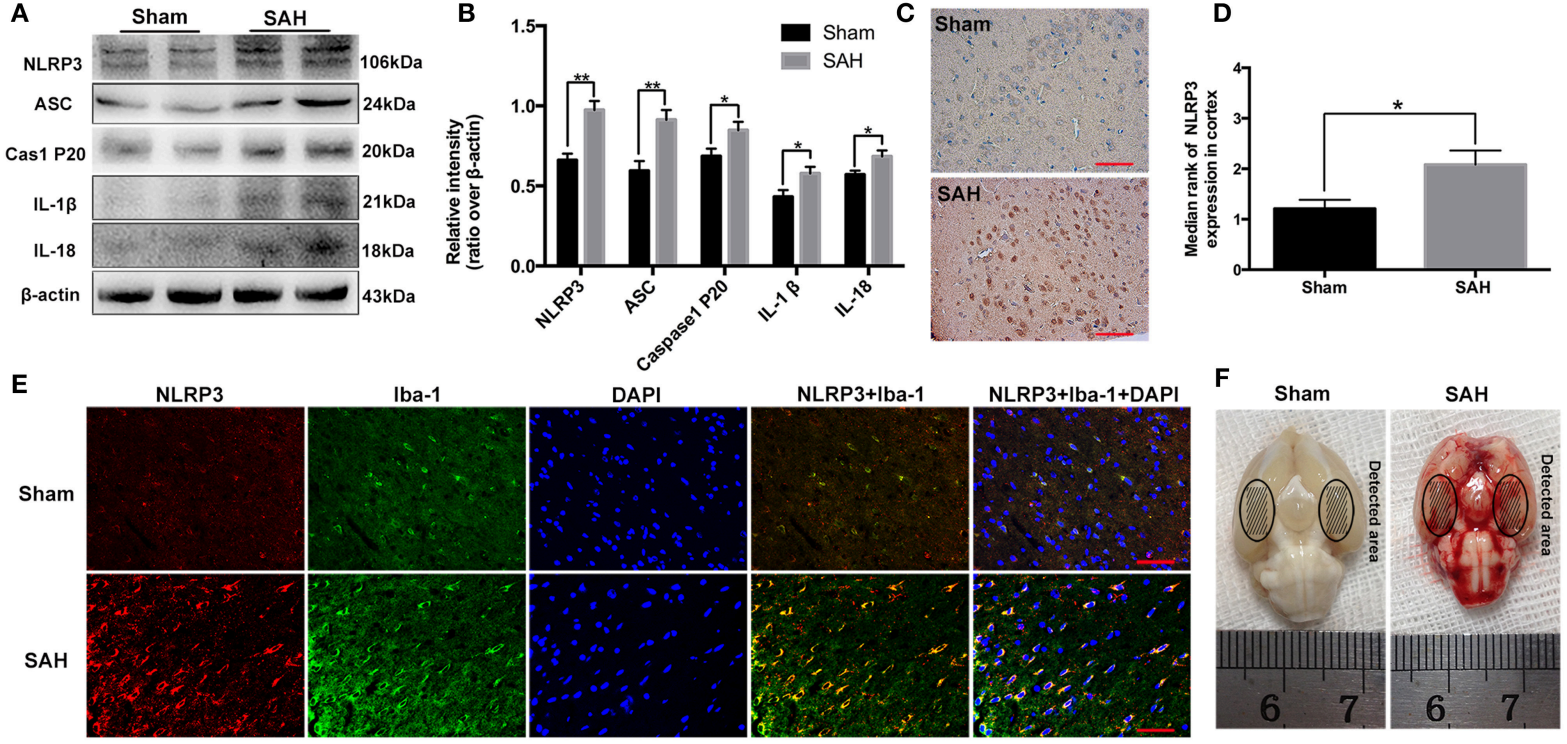
Expression and cellular distribution of the NLRP3 inflammasome components in the basal cortex at 24 h post-subarachnoid hemorrhage (SAH). **(A)** Western blot assay for the expression of NLRP3, ASC, caspase-1 P20 subunit, IL-1β and IL-18; **(B)** Quantification of NLRP3, ASC, caspase-1 P20 subunit, IL-1β and IL-18 expression is shown; **(C)** Representative photomicrographs of NLRP3 immunohistochemistry in the sham and 24 h post-SAH groups; **(D)** Quantification of the NLRP3 immunohistochemistry; **(E)** Representative photographs of NLRP3 immunofluorescence staining in microglia in the basal cortex at 24 h following SAH; **(F)** Schematic representation of the areas taken for assay. Bars represent the mean ± SEM. ^**^P < 0.01, ^*^P < 0.05, and ns means non-significant. Scale Bars = 50 μm.

*In vivo* experiments, the prechiasmatic cistern injection model was prepared according to previous studies (Figure 1F) (Li J. et al., 2015; Yan et al., 2015; Zhang X. S. et al., 2015). Briefly, after the rats were anesthetized with 10% chloral hydrate (0.35 ml/100 g), a total of 0.35 ml of non-heparinized fresh autologous arterial blood from the femoral artery was slowly (in the course of 20 s) injected into the prechiasmatic cistern under aseptic conditions (Zhang X. S. et al., 2015). After the procedures, the animals were kept in a 30°, heads-down position for 20 min. Rats were returned to their feeding room after wake up from anesthesia.

*In vitro* experiments, primary cultured cortical neurons were stimulated with oxyhemoglobin (oxyHb, Ruibio, O7109). Primary cultured neurons were prepared as mentioned above. oxyHb was resolved into 10 μM with culture medium, which was determined according to previous study (Wang Z. et al., 2016). Neurons were stimulated with 10 μM oxyHb for 24 h to mimic SAH condition.

### Study design

#### Experiment 1

For *in vivo* experiments, rats were randomly assigned to the various groups: sham group (*n* = 36); sham + 60 mg/kg RSV group (*n* = 36); SAH group (*n* = 12); SAH + vehicle group (*n* = 36); SAH + 30 mg/kg RSV group (*n* = 12), SAH + 60 mg/kg RSV group (*n* = 36), and SAH + 90 mg/kg RSV group (*n* = 12). All rats were sacrificed to collect brain samples at 24 h after SAH. Post-treatment assessments included neurological scores, brain edema, Western blot analysis, double immunofluorescence staining, and TUNEL staining.

#### Experiment 2

For *in vitro* experiments, primary cultured cortical neurons were randomly divided into six groups: control group, control + 20 μM RSV group, SAH + vehicle group, SAH + 1 μM RSV group, SAH + 10 μM RSV group, and SAH + 20 μM RSV group. The neurons were collected for Western blot analysis, cell viability analysis, and histopathological study.

### Drug administration

For *in vivo* experiments, RSV (Sigma-Aldrich, St. Louis, MO, USA) was dissolved in 1% dimethylsulfoxide (DMSO) and physiological saline. In the rats of sham + RSV or SAH + RSV groups, RSV was injected intraperitoneally at different doses (30, 60, 90 mg/kg) at 2 and 12 h post-injury (Zhang et al., 2016a). The doses were determined based on previous studies (Shao et al., 2014; Zhang et al., 2016a). Rats in SAH + vehicle group received an intraperitoneal injection of the same volume of vehicle at the corresponding time point. For *in vitro* experiments, RSV was dissolved in DMSO and then added to neuronal medium to reach different final concentrations (1 μM, 10 μM, and 20 μM). The concentrations of RSV used in *in vitro* study were based on previous studies *in vitro* (Wang et al., 2015; Zhang Q. et al., 2015).

### Western blot

Western blot was carried out as previously described (Zhang X. S. et al., 2015). Briefly, protein samples were separated by 10% SDS-PAGE and electrophoretically transferred to polyvinylidene-difluoride (PVDF) membrane (Bio-Rad Lab, Hercules, CA, USA). After the PVDF membrane was blocked for 2 h, the membrane was then incubated with primary antibodies overnight (Zhang X. S. et al., 2015). The primary antibodies against NLRP3 (cat# SC-66846), ASC (cat# SC-22514), caspase-1 (cat# SC-398715), IL-1β (cat# SC-32294), IL-18 (cat# SC-7954), TNF-α (cat# SC-52746), Bcl-2 (cat# SC-492), and Bax (cat# SC-526) were bought from Santa Cruz Biotechnology. Antibody against caspase-3 (cat# 9661) was purchased from Cell Signaling Technology. After that, the membranes were incubated in the appropriate horseradish peroxidase (HRP)-conjugated secondary antibody. The blotted protein bands were developed using enhanced chemiluminescence kit (Amersham, Arlington Heights, IL, USA).

### Immunohistochemistry

Briefly, brain sections (4 μm thickness) were incubated overnight at 4°C with primary antibody against NLRP3, ASC, and caspase-1. After washing carefully in PBS for about 15 min, the sections were then incubated with HRP-conjugated secondary antibody for 1 h at room temperature. 3,3′-diaminobenzidine (DAB) was used to visualize NLRP3, ASC, and caspase-1. The intensity of staining in each section was evaluated by using a five-grades scoring system (Wang et al., 2012; Zhang X. S. et al., 2015). “0” indicates that there were no detectable positive cells; “1” represents very low density of positive cells; “2” represents a moderate density of positive cells; “3” represents the higher, but not maximal density of positive cells; and “4” represents the highest density of positive cells (Wang et al., 2012; Zhang et al., 2016a).

### Immunofluorescence staining

Immunofluorescence staining was carried out as described previously (Sun et al., 2014). Frozen tissue sections (6 μm thickness) were incubated in blocking buffer for 2 h at room temperature. After they were washed three times with PBS, sections were incubated with primary antibodies and corresponding secondary antibodies. After washing with PBS for three times, the slides were stained with 4-diamidino-2-phenylindole (DAPI) for 2 min. Images were obtained using a ZEISS HB050 inverted microscope system and the fluorescently stained cells were analyzed using Image J program.

### TUNEL staining

Terminal deoxynucleotidyl transferase-mediated dUTP nick-end labeling (TUNEL) staining was performed according to the manufacturer's instruction (Roche, South San Francisco, CA, USA). In brief, after brain sections or cultured neurons were incubated with primary antibody at 4°C overnight, the slides or cultured neurons were incubated with TUNEL reaction mixture for 1 h at 37°C. The slides were washed several times with PBS and then stained with DAPI for 2 min. The quantification of TUNEL-positive neurons was performed by a pathologist blinded to the experiments groups.

### Neurological scores and brain water content

The neurological scores were recorded 24 h after SAH with a 6-point scoring system (Table 1; Zhang et al., 2016a). Brain water content was evaluated as previously reported (Zhang et al., 2016a). Briefly, brains were removed and separated into cerebrum, cerebellum and brain stem. Each part was weighed immediately to obtain the wet weight and dried for 72 h at 100°C to obtain the dry weight. The percentage of water content was calculated as follows: [(wet weight-dry weight)/wet weight] × 100% (Zhang et al., 2016a).

**Table 1:** Behavior scores.

**Category**	**Behavior**	**Score**
Appetite	Finished meal	0
	Left meal unfinished	1
	Scarcely ate	2
Activity	Walk and reach at least three corners of the cage	0
	Walk with some stimulation	1
	Almost always lying down	2
Deficits	No deficits	0
	Unstable walk	1
	Impossible to walk	2

### Cell viability analysis

The primary cultured neurons viability was evaluated by measuring the lactate dehydrogenase (LDH) activity. LDH activity was determined with an assay kit according to the manufacturers' protocol (Beyotime Biotechnology, Shanghai, China).

### Statistical analysis

Data were presented as mean ± S.E.M. SPSS 19.0 was used for statistical analysis of the data. Statistical analysis between two groups were performed with the Student's *t*-test and between multiple groups with one-way analysis of variance (ANOVA) followed by Tukey *post-hoc* test. Statistical significance was inferred at *P* < 0.05.

## Results

### General observations and mortality rate

There were no statistical differences in mean arterial blood pressure and heart rate among all experimental groups. No animals died in the sham group (0/36 rats) and sham + 60 mg/kg RSV group (0/36 rats). The mortality rate of the rats was 25.0% (3/12 rats) in the SAH group; 19.4% (7/36 rats) in the SAH + vehicle group; 25% (3/12 rats) in the SAH + 30 mg/kg RSV group; 13.9% (5/36 rats) in the SAH + 60 mg/kg RSV group; 16.7% (2/12 rats) in the SAH + 90 mg/kg RSV group.

### NLRP3 inflammasome components were upregulated at 24 h post-SAH

Western blot results showed that NLRP3 components, including NLRP3 (0.66 ± 0.04 vs. 0.98 ± 0.06), ASC (0.59 ± 0.06 vs. 0.91 ± 0.06), caspase-1 (0.69 ± 0.05 vs. 0.85 ± 0.05), IL-1β (0.43 ± 0.04 vs. 0.58 ± 0.04), and IL-18 (0.57 ± 0.03 vs. 0.69 ± 0.04) were evidently increased at 24 h post-SAH (Figures 1A,B). The distribution of NLRP3 in the brain cortex was further identified by immunohistochemistry and immunofluorescence staining (Figures 1C–E). As shown, NLRP3 was weakly expressed in the sham group. In contrast, NLRP3 was evidently increased in the cortex at 24 h post-SAH (1.21 ± 0.18 vs. 2.08 ± 0.28), and the increased NLRP3 was mainly located in microglia after SAH.

### Effects of RSV on the NLRP3 inflammasome signaling pathway

**Figure 2:**
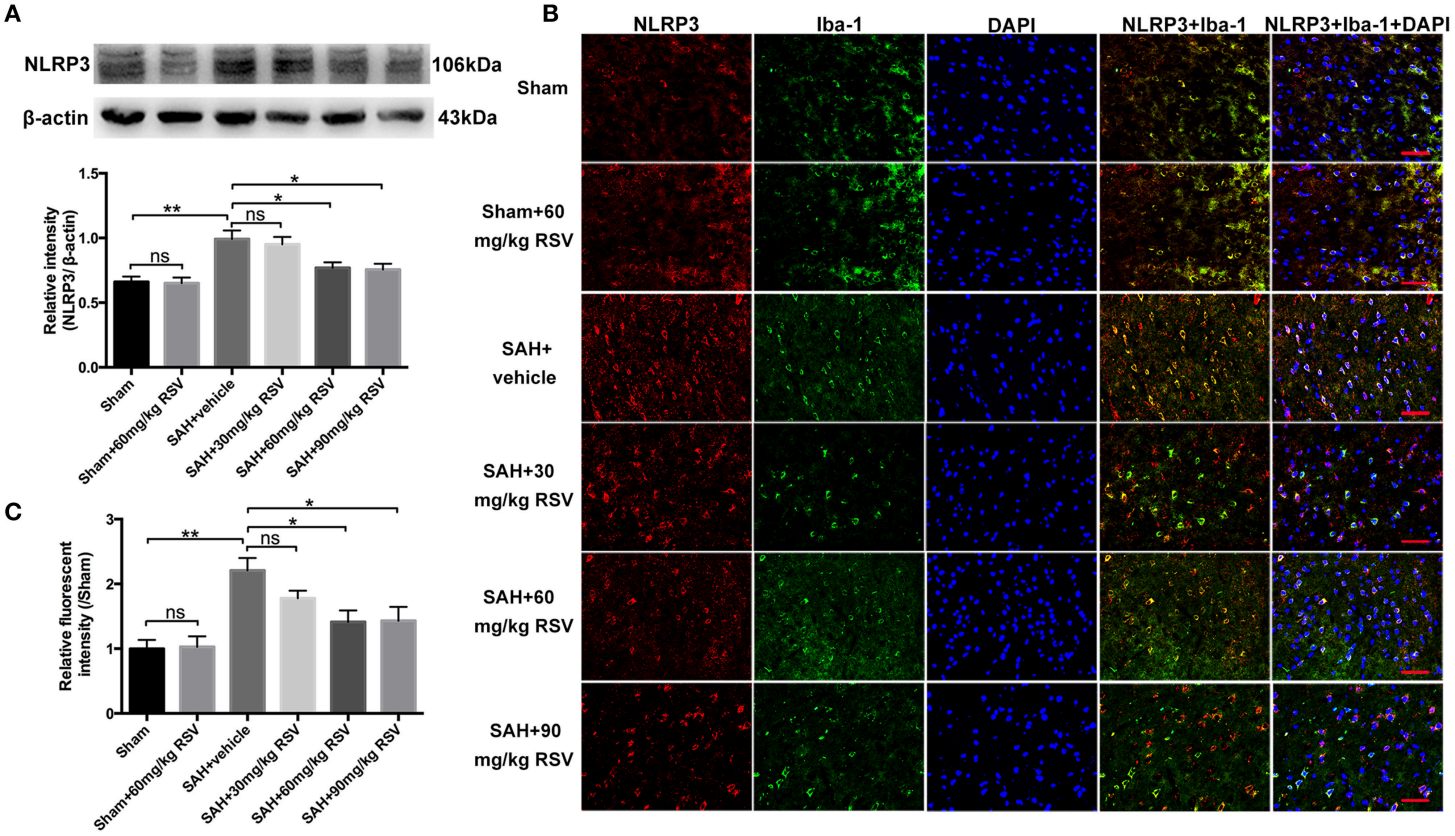
Effects of different doses of RSV on the expression of NLRP3 at 24 h post-SAH. **(A)** Western blot analysis for NLRP3 expression in all different groups; **(B)** Representative photographs of NLRP3 immunofluorescence staining in microglia in all groups; **(C)** Quantification of the NLRP3 immunofluorescence staining in different groups. Bars represent the mean ± SEM. ^**^P < 0.01, ^*^P < 0.05, and ns means non-significant. Scale Bars = 50 μm.

As shown in Figure 2A, both 60 mg/kg and 90 mg/kg RSV treatment significantly reduced the enhanced expression of NLRP3 in brain cortex after SAH (0.99 ± 0.07 vs. 0.77 ± 0.04, 0.99 ± 0.07 vs. 0.76 ± 0.05). However, 30 mg/kg RSV treatment did not inhibit the expression of NLRP3 when compared with that in the SAH + vehicle group (0.99 ± 0.07 vs. 0.95 ± 0.06). In addition, we noted that there was no statistical difference in the expression of NLRP3 between the SAH + 60 mg/kg RSV and SAH + 90 mg/kg RSV groups (0.77 ± 0.04 vs. 0.76 ± 0.05). Immunofluorescence staining results (Figures 2B,C) was similar to that of Western blot, suggesting that 60 mg/kg RSV treatment could efficiently inhibit NLRP3 expression after SAH. Therefore, we chose 60 mg/kg RSV in the following experiments.

**Figure 3:**
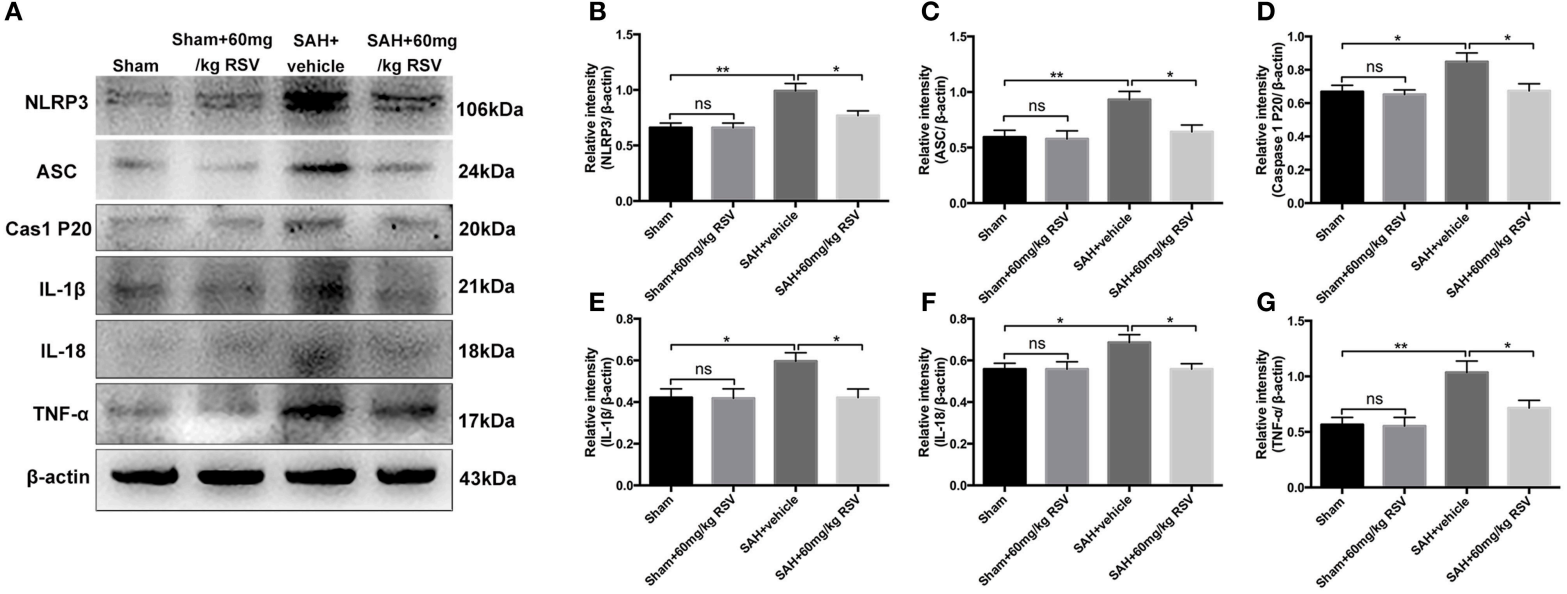
Effects of RSV on the activation of NLRP3 inflammasome activation-related pathway at 24 h post-SAH. **(A)** Representative images of Western blot for NLRP3, ASC, caspase-1 P20 subunit, IL-1β, IL-18, and TNF-α; **(B–G)** Quantitative analyses of NLRP3, ASC, caspase-1 P20 subunit, IL-1β, IL-18, and TNF-α among all experimental groups, respectively. Bars represent the mean ± SEM. ^**^P < 0.01, ^*^P < 0.05, and ns means non-significant.

**Figure 4:**
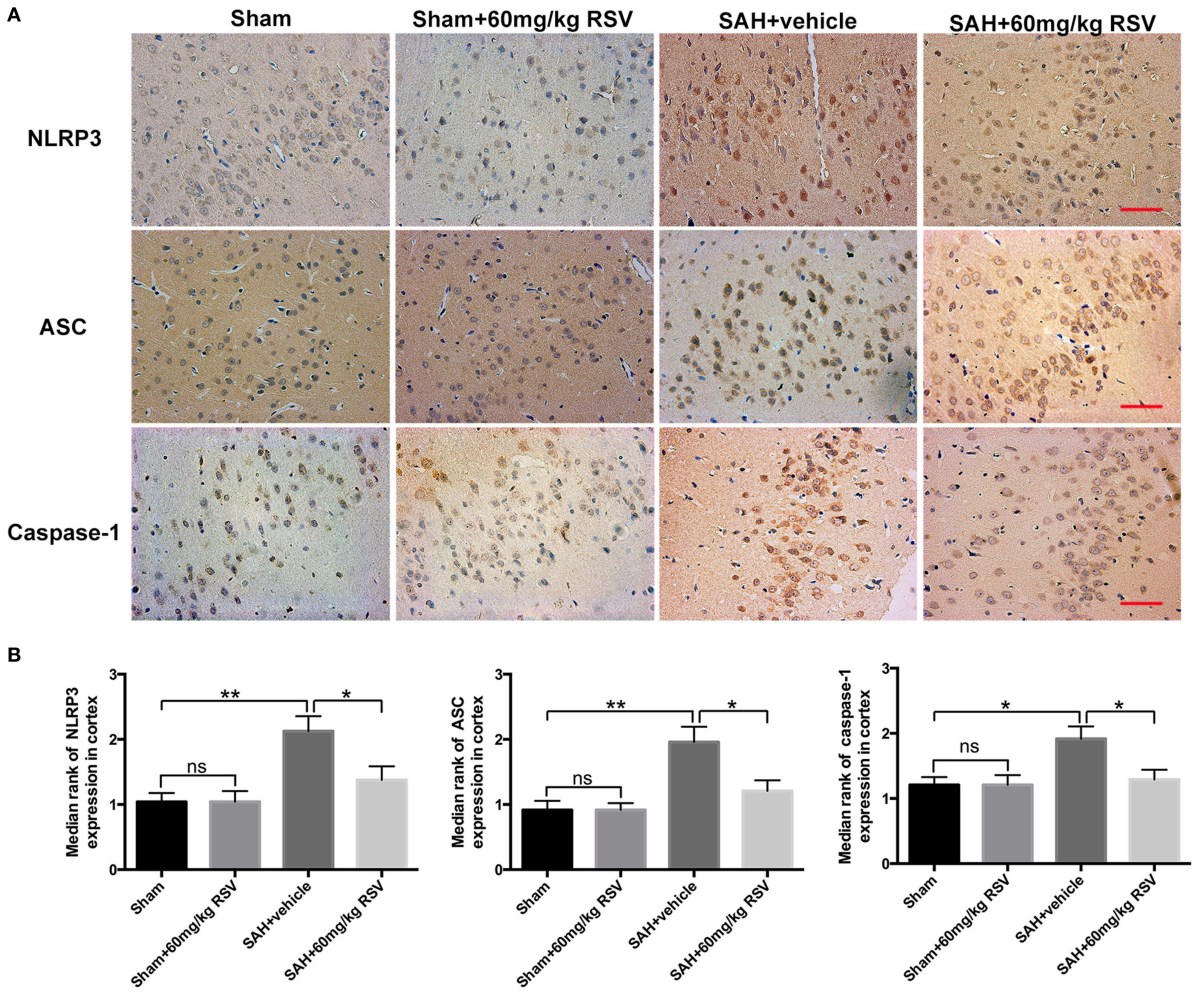
Effects of RSV on the NLRP3 inflammasome components distribution at 24 h post-SAH. **(A)** Representative images of NLRP3, ASC, and caspase-1 P20 subunit immunohistochemistry in all groups; **(B)** Quantification of NLRP3, ASC, and caspase-1 P20 subunit immunohistochemistry in all experimental groups. Bars represent the mean ± SEM. ^**^P < 0.01, ^*^P < 0.05, and ns means non-significant. Scale Bars = 50 μm.

We next evaluated the effects of RSV on the NLRP3 inflammasome signaling pathway. Western blot results (Figures 3A–G) showed that the protein expressions of NLRP3 (0.66 ± 0.04 vs. 0.99 ± 0.07), ASC (0.59 ± 0.06 vs. 0.93 ± 0.07), caspase-1 (0.67 ± 0.04 vs. 0.85 ± 0.05), IL-1β (0.42 ± 0.04 vs. 0.60 ± 0.04), IL-18 (0.56 ± 0.03 vs. 0.69 ± 0.04), and TNF-α (0.57 ± 0.07 vs. 1.04 ± 0.10) were markedly upregulated at 24 h after SAH. In contrast, administration of RSV significantly reduced the expression of NLRP3 inflammasome components as compared with the SAH + vehicle group (0.99 ± 0.07 vs. 0.77 ± 0.04, 0.93 ± 0.07 vs. 0.64 ± 0.06, 0.85 ± 0.05 vs. 0.67 ± 0.04, 0.60 ± 0.04 vs. 0.42 ± 0.04, 0.69 ± 0.04 vs. 0.56 ± 0.03, 1.04 ± 0.10 vs. 0.72 ± 0.07). Similarly, immunohistochemistry (Figures 4A,B) also revealed elevated NLRP3, ASC, and caspase-1 immunoreactivities in the brain cortex after SAH, which were also reversed by RSV treatment (2.13 ± 0.23 vs. 1.38 ± 0.21, 1.96 ± 0.24 vs. 1.21 ± 0.16, 1.92 ± 0.19 vs. 1.29 ± 0.15). These results suggested that RSV could inhibit NLRP3 inflammasome activation after SAH.

### Effects of RSV on microglia activation and neutrophils infiltration at 24 h post-SAH

**Figure 5:**
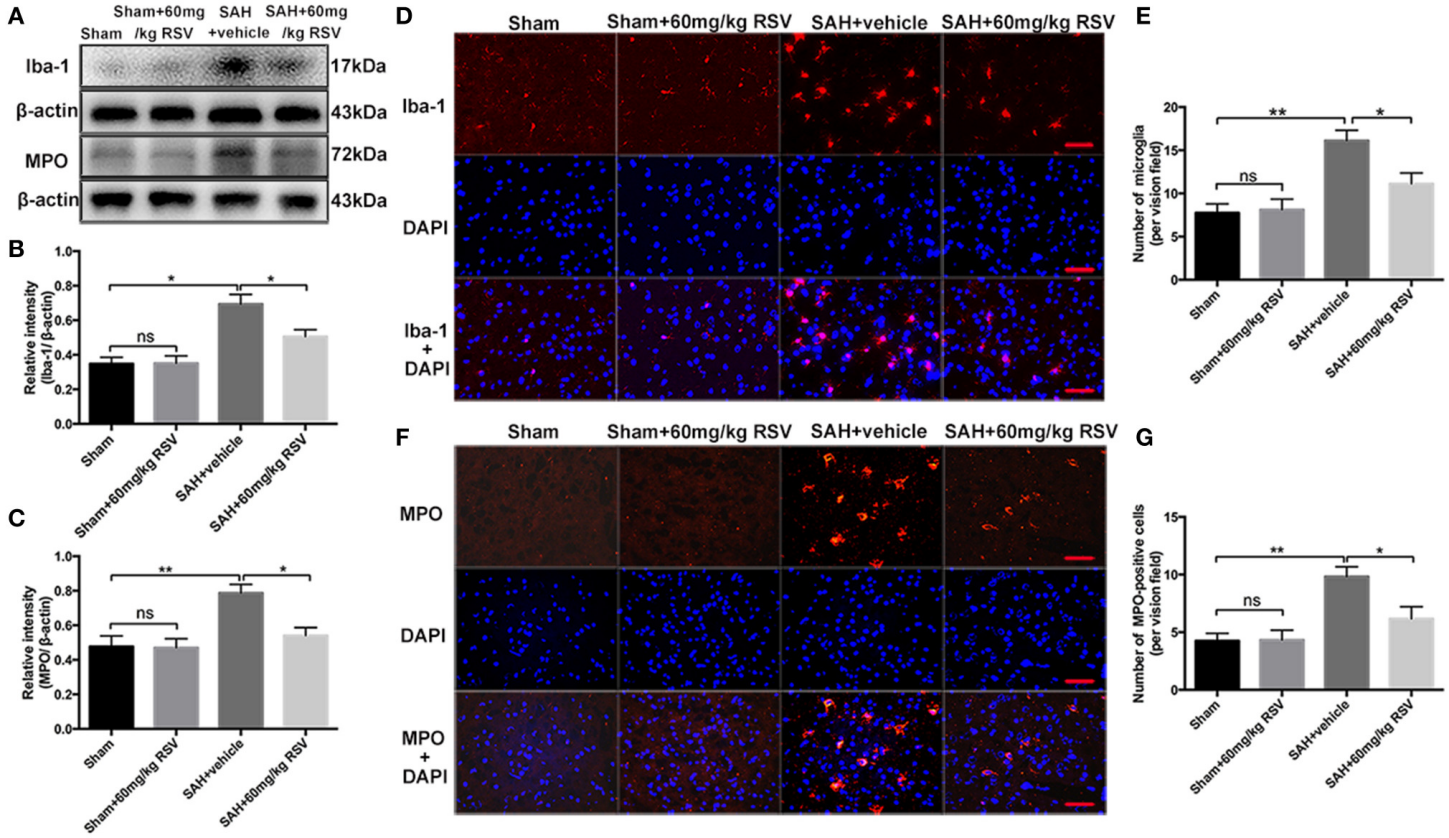
Effects of RSV treatment on microglia activation and neutrophils infiltration at 24 h post-SAH. **(A)** Western blot assay for the Iba-1 and MPO levels in the brain samples at 24 h after SAH; **(B,C)** Quantitative analyses of Iba-1 and MPO among all experimental groups; **(D,F)** Representative photomicrographs of Iba-1 and MPO immunofluorescence staining in different groups at 24 h following SAH; **(E,G)** Quantification the number of Iba-1- and MPO- positive cells in all groups. Bars represent the mean ± SEM. ^**^P < 0.01, ^*^P < 0.05, and ns means non-significant. Scale Bars = 50 μm.

Activation of microglia and neutrophils infiltration play important roles in inflammatory response after SAH. Our results showed that the expressions of Iba-1 (0.35 ± 0.04 vs. 0.69 ± 0.06) and MPO (0.48 ± 0.06 vs. 0.79 ± 0.05) were higher at 24 h after SAH insults than that in the sham group (Figures 5A–C). In addition, the number of Iba-1- and MPO-positive cells in the brain cortex was also significantly enhanced after SAH (7.75 ± 1.04 vs. 16.13 ± 1.21, 4.25 ± 0.66 vs. 9.83 ± 0.84) (Figures 5D–G). After administration with RSV, the upregulated Iba-1 (0.69 ± 0.06 vs. 0.51 ± 0.04) and MPO (0.79 ± 0.05 vs. 0.54 ± 0.05) expression as well as the Iba-1- (16.13 ± 1.21 vs. 11.13 ± 1.24) and MPO-positive (9.83 ± 0.84 vs. 6.17 ± 1.05) cells were markedly reduced when compared with the SAH + vehicle group (Figures 5A–G). These results suggested that RSV could inhibit inflammatory response in the brain after SAH.

### Influence of RSV on neuronal apoptosis, brain water content, and neurobehavioral impairment at 24 h after SAH

**Figure 6:**
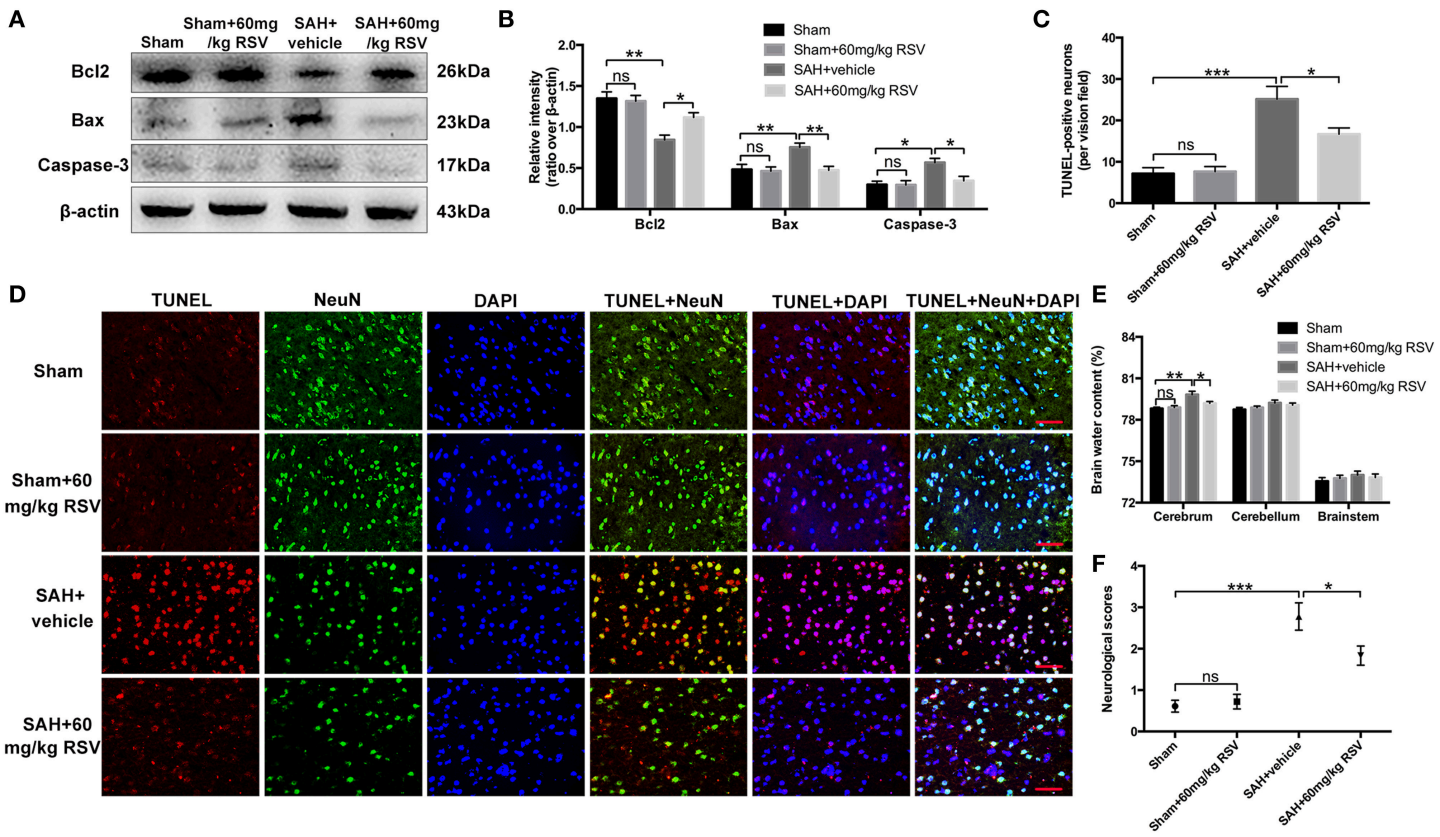
Effects of RSV on neuronal cell apoptosis, brain edema, and neurological function at 24 h following SAH. **(A)** Western blot assay for the expression of Bcl2, Bax and caspase-3 in the groups; **(B)** Quantification of the protein levels of Bcl2, Bax and caspase-3; **(C)** Quantification of TUNEL-positive neurons in all groups; **(D)** Representative photomicrographs of TUNEL staining in different groups; **(E,F)** Quantification of brain water content and neurological functions in different groups. Bars represent the mean ± SEM. ^***^P < 0.001, ^**^P < 0.01, ^*^P < 0.05, and ns means non-significant. Scale Bars = 50 μm.

Western blot results showed that RSV treatment significantly reduced Bax (0.75 ± 0.05 vs. 0.47 ± 0.05) and caspase-3 (0.57 ± 0.05 vs. 0.34 ± 0.05) protein levels, and enhanced the diminished level of Bcl2 (0.85 ± 0.06 vs. 1.11 ± 0.06) compared with the SAH + vehicle group (Figures 6A,B). TUNEL staining further showed that RSV administration could increase the proportion of surviving neurons after SAH (25.15 ± 3.07 vs. 16.67 ± 1.48) (Figures 6C,D). Brain water content and neurobehavioral function were blindly investigated at 24 h following SAH. As shown (Figures 6E,F), brain edema and neurological impairment were significantly aggravated after SAH insults compared with the sham group (0.788 ± 0.098 vs. 0.798 ± 0.240, 0.61 ± 0.14 vs. 2.78 ± 0.33). In contrast, RSV treatment could partly reverse the aggravated brain edema and neurological impairment after SAH (0.798 ± 0.240 vs. 0.792 ± 0.127, 2.78 ± 0.33 vs. 1.83 ± 0.23).

### Effects of RSV on neuronal damage in primary cortical neurons *in vitro*

**Figure 7:**
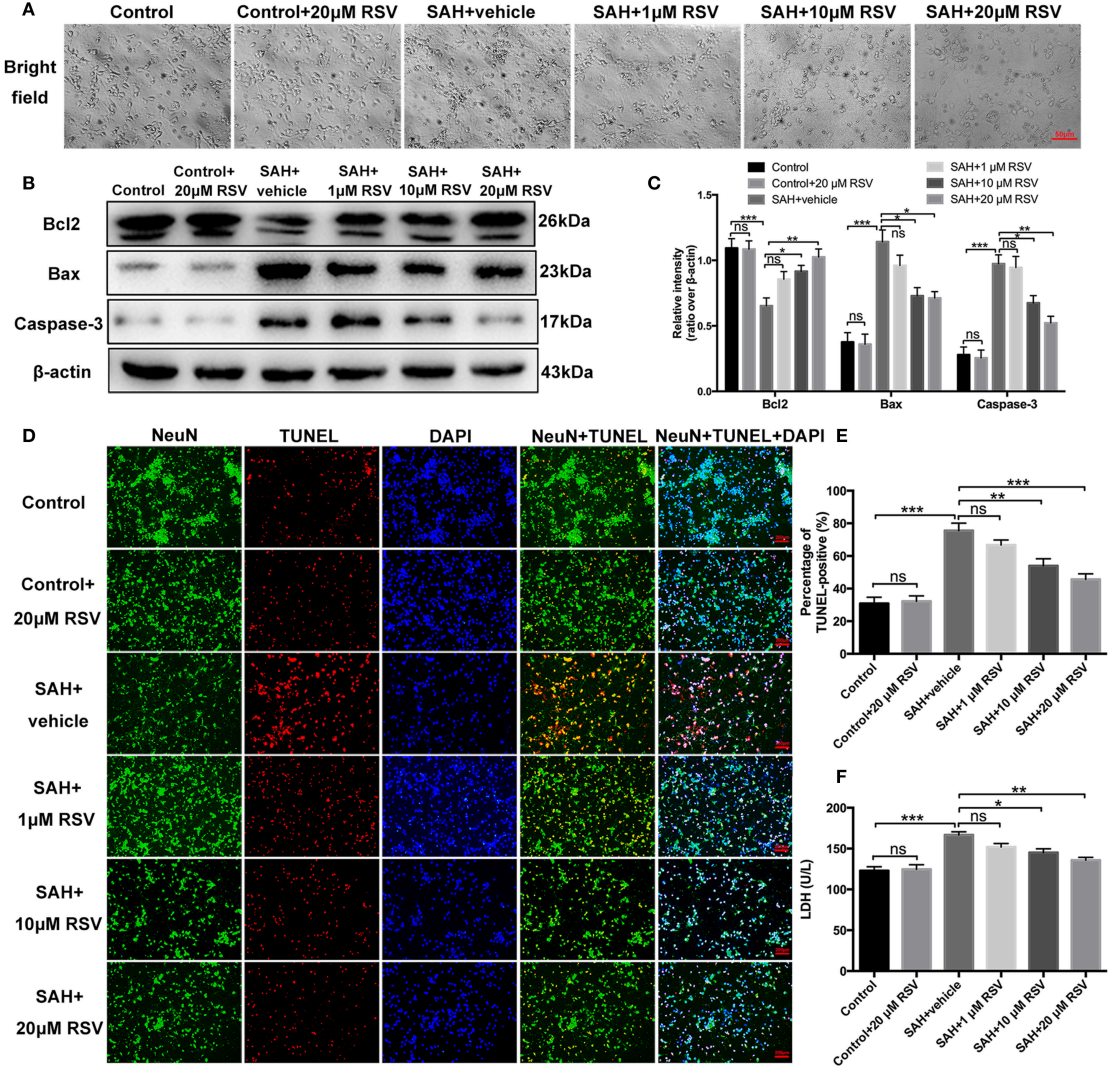
Effects of RSV on neuronal damage and cell viabilities in primary cortical neurons exposed to oxyHb. **(A)** Representative images of light microscopic for primary cortical neurons in all different groups; **(B)** Western blot assay for the apoptotic pathways; **(C)** Quantification of the protein levels of Bcl2, Bax and caspase-3 in all groups; **(D)** Representative photomicrographs of TUNEL staining in different groups; **(E)** Quantification of TUNEL-positive neurons in all groups; **(F)** Quantitative analyses of LDH activity in all groups. Bars represent the mean ± SEM. ^***^P < 0.001, ^**^P < 0.01, ^*^P < 0.05, and ns means non-significant.

As shown in Figure 7A, neurons exposed to oxyHb showed evident neuronal degeneration characterized by swollen neuronal cell bodies with loss of synapses and brightness. Compared with SAH + vehicle group, RSV treatment significantly prevented these morphological changes in a dose-dependent manner. We then evaluated the influence of different doses of RSV on apoptotic pathways, TUNEL-apoptosis, and LDH release in primary cortical neurons (Figures 7B–F). Results showed that 10 or 20 μM RSV treatment evidently inhibited apoptotic pathways, reduced the number of TUNEL-positive neurons (75.51 ± 4.60 vs. 53.97 ± 4.31, 75.51 ± 4.60 vs. 45.60 ± 3.44), and decreased LDH activity (166.75 ± 3.87 vs. 145.12 ± 4.57, 166.75 ± 3.87 vs. 135.67 ± 3.60) after oxyHb stimulation in a dose-dependent manner. However, 1 μM RSV treatment did not alleviate neuronal damage when compared with SAH + vehicle group. One possible reason could be that 1 μM RSV might not be sufficient to produce protection against neuronal damage exposed to oxyHb. These results suggested that RSV treatment could also reduce neuronal damage in SAH model *in vitro*.

## Discussion

In the current study, we evaluated the NLRP3 inflammasome activation responded to brain injury following SAH, and its relationship with the RSV's neuroprotective effects in experimental SAH models. The major findings were as follows: (1) NLRP3 inflammasome was upregulated in the brain cortex after SAH, and the enhanced NLRP3 after SAH was mainly expressed in microglia; (2) RSV treatment inhibited NLRP3 inflammasome activation after SAH, and suppressed the microglia activation and neutrophils infiltration; (3) administration with RSV reduced neuronal cell apoptosis, brain edema, and neurological impairment after SAH; (4) RSV significantly alleviated neuronal damage, prevented neurodegeneration, and improved cell viabilities in primary cortical neurons with a dose-dependent manner.

Inflammation has been proved to play key roles in the pathophysiology of SAH-induced brain injury (Zhang et al., 2016a,b; Wu Q. et al., 2017). At the onset of SAH, the infiltration of blood-borne leukocyts is one of the earliest events (Ma et al., 2014). Microglia, the primary resident macrophages of the CNS, can be activated by the infiltrated leukocytes after SAH. Subsequently, the infiltrated leukocytes and activated microglia produce and release a number of pro-inflammatory cytokines to cause direct damage to surrounding neural cells and amplify leukocyte recruitment to further aggravate brain damage after SAH (Chen et al., 2013). However, the mechanisms by which SAH stimulates the inflammatory response have not been fully elucidated.

Recently, there is a wealth of studies have shown that inflammasome is involved in the pathological process of various sterile inflammatory disorders, such as gout, Crohn disease, systemic-onset juvenile arthritis, and cryopyrin-associated periodic syndromes (Walsh et al., 2014; Coll et al., 2015; Zhu et al., 2017). The inflammasome is a large multiple protein complex that modulates maturation and release of the pro-inflammatory cytokines (Chen et al., 2013). Among all inflammasomes, the NLRP3 inflammasome is the best studied from this family (Ma et al., 2014; Guo et al., 2016). The NLRP3 inflammasome contains NLR protein cryopyrin, the adapter ASC (apoptosis-associated speck-like protein containing a caspase recruitment domain), and pro-caspase-1(Wang Y. et al., 2016). Upon activation, aberrant NLRP3 inflammasome results in a robust release of mature IL-1β and IL-18, which plays key roles in a variety of diseases, including CNS disorders (Chen et al., 2013; Ma et al., 2014; Coll et al., 2015).

In experimental ischemic stroke models, Yang et al reported that NLRP3 deficiency alleviated neurovascular damage via attenuation of inflammatory pathways (Yang et al., 2014). Ma et al also reported that NLRP3 inflammasome amplified the inflammatory response following experimental intracerebral hemorrhage (ICH) model, and NLRP3 knockdown reduced inflammatory damage and improved neurological functions following ICH (Ma et al., 2014). Notably, recent studies have observed that NLRPP3 inflammasome contributes to inflammatory damage following SAH, and inhibition NLRP3 inflammasome by hydrogen-rich saline, melatonin and minocycline could ameliorate EBI after SAH (Chen et al., 2013; Li J. et al., 2015; Shao et al., 2015; Dong et al., 2016). In the current study, we also observed that the NLRP3 inflammasome components were upregulated after SAH. In addition, the enhanced NLRP3 after SAH was mainly distributed in microglia. These results were consistent with previous studies suggesting that NLRP3 inflammasome may play a pivotal role in the pathologic process of SAH and inhibiting NLRP3 inflammasome activation might be a useful therapeutic intervention following SAH (Chen et al., 2013; Li J. et al., 2015).

RSV is a natural polyphenolic compound distributed at high concentrations in grapes, pines, as well as peanuts (Zhou et al., 2014). Recently, a number of studies have highlighted that RSV may be a promising agent in a number of CNS disorders due to its multiple functions, such as anti-inflammatory, anti-oxidant, and neuroprotective effects (Shao et al., 2014; Arteaga et al., 2015; Lopez et al., 2015; Zhang et al., 2016a). RSV has been demonstrated to ameliorate neurovascular damage in ischemic stroke and TBI (Della-Morte et al., 2009; Lin et al., 2014; Koronowski et al., 2015). Importantly, RSV also has beneficial effects against SAH insults via its powerful anti-inflammatory action (Shao et al., 2014; Zhang et al., 2016a). However, the molecular mechanisms underlying the neuroprotective effects of RSV in SAH remain elusive. Previous studies reported that RSV could modulate NLRP3 inflammasome activation in several different research fields (Fu et al., 2013; Yang and Lim, 2014; Sui et al., 2016). Yang et al proved that RSV ameliorated hepatic metaflammation by inhibiting NLRP3 inflammasome activation (Yang and Lim, 2014). Sui et al found that RSV could protect against sepsis-associated encephalopathy via inhibiting NLRP3/IL-1β axis in microglia (Sui et al., 2016). Thus, we speculated that RSV could protect the brain against SAH insults through the inhibition of NLRP3 inflammasome activation.

Our findings were consistent with previous studies (Fu et al., 2013; Yang and Lim, 2014; Sui et al., 2016). We found that RSV treatment markedly inhibited the NLRP3 inflammasome activation and the subsequent production of pro-inflammatory cytokines after SAH. In addition, microglia activation and neutrophil infiltration, which played important roles in cerebral inflammation after SAH, were also suppressed by RSV administration. Cell apoptosis is a key element of EBI after SAH and is responsible for disastrous outcomes following SAH (Sehba et al., 2012; Zhang X. S. et al., 2015). In addition to the powerful anti-inflammatory function, the anti-apoptotic property is also an important part of RSV's protection (Lopez et al., 2015). In the present study, RSV also showed neuroprotective effects via inhibition of neuronal apoptosis. These might be mediated by RSV's anti-inflammatory and anti-apoptosis functions. To further determine the beneficial effects of RSV on neuronal damage after SAH, we evaluated the influence of RSV on primary cortical neurons exposed to oxyHb. As expected, RSV evidently prevented neuronal degeneration, inhibited neuronal apoptosis, and restored cell viabilities in primary cortical neurons in a dose-dependent manner, suggesting that RSV was an efficient neuroprotective drug against SAH injury. These findings further support that NLRP3 inflammasome activation plays a key role in the development of EBI after SAH. Meanwhile, the NLRP3 inflammasome inhibition contributed to the profound neuroprotective effects of RSV against EBI induced SAH.

It should be reported that our study has several limitations. Firstly, recent studies reported that microglia have dual phenotypes, pro-inflammatory M1 and anti-inflammatory M2 phenotypes. M1 microglia can increase pro-inflammatory cytokines and upregulate reactive oxygen and nitrogen species. In contrast, M2 microglia release anti-inflammatory cytokines and growth factors, exerting the opposite effects of M1 microglia (Lan et al., 2017b; Yang et al., 2017; Zhang et al., 2017). Studies have shown that activated microglia develop into different phenotypes in different microenvironments and exhibit distinct functions (Lan et al., 2017a; Zhang et al., 2017). In this study, we showed that RSV inhibited microglia activation after SAH. However, we are not sure whether RSV can modulate M2 microglia activation in SAH. Yang et al. reported that RSV could reduce inflammatory damage and promoted microglia polarization to the M2 phenotype in LPS-induced neuroinflammation (Yang et al., 2017). Thus, we speculated that RSV could inhibit microglia activation by promoting microglia polarization toward to the M2 phenotype in SAH. Secondly, the molecular basis underlying NLRP3 inflammasome activation induced by SAH is far from complete. In addition, how RSV regulates NLRP3 inflammasome activation in SAH still remains obscure. Previous studies demonstrated that reactive oxygen species (ROS) was a necessary step for NLRP3 activation, and inhibition ROS production by ROS inhibitor suppressed NLRP3 inflammasome activation (Murphy, 2009). Considering that oxidative damage is a crucial component in EBI pathogenesis, and RSV has a strong anti-oxidant property in other research fields (Zhang X. S. et al., 2014; Zhou et al., 2014; Narayanan et al., 2015; Zhang et al., 2016b), we speculated that RSV might inhibit NLRP3 inflammasome activation by suppressing ROS production. Finally, we could not exclude the possible effects of other pathways participated in the beneficial function of RSV in SAH. Given that the present study is a preliminary research, additional works are still warranted to clarify these issues.

## Conclusion

In summary, our study demonstrated that RSV could protect against EBI, at least partly via inhibiting the NLRP3 inflammasome signaling pathway-related inflammatory response. Although more work is required, current data shed new light on the treatment of SAH, and suggest that RSV may be an attractive therapeutic agent in treatment of SAH.

## Author contributions

XiaZ and QW wrote the paper. XiaZ, QW, YL, JL, and SL performed the experiments. XiaZ and WL contributed to the analysis of samples and data. QZ, MZ, CH, and XinZ designed the study and revised the manuscript. All authors approved the final version of the manuscript.

### Conflict of interest statement

The authors declare that the research was conducted in the absence of any commercial or financial relationships that could be construed as a potential conflict of interest.

## References

[B1] Arteaga O. Revuelta M. Uriguen L. Alvarez A. Montalvo H. Hilario E. (2015). Pretreatment with resveratrol prevents neuronal injury and cognitive deficits induced by perinatal hypoxia-ischemia in rats. PLoS ONE 10:e0142424. 10.1371/journal.pone.0142424 26544861 PMC4636303

[B2] Chen S. Feng H. Sherchan P. Klebe D. Zhao G. Sun X.. (2014). Controversies and evolving new mechanisms in subarachnoid hemorrhage. Prog. Neurobiol. 115, 64–91. 10.1016/j.pneurobio.2013.09.002 24076160 PMC3961493

[B3] Chen S. Ma Q. Krafft P. R. Hu Q. Rolland W. II. Sherchan P.. (2013). P2X7R/cryopyrin inflammasome axis inhibition reduces neuroinflammation after SAH. Neurobiol. Dis. 58, 296–307. 10.1016/j.nbd.2013.06.011 23816751 PMC3771387

[B4] Coll R. C. Robertson A. A. Chae J. J. Higgins S. C. Munoz-Planillo R. Inserra M. C.. (2015). A small-molecule inhibitor of the NLRP3 inflammasome for the treatment of inflammatory diseases. Nat. Med. 21, 248–255. 10.1038/nm.3806 25686105 PMC4392179

[B5] Della-Morte D. Dave K. R. DeFazio R. A. Bao Y. C. Raval A. P. Perez-Pinzon M. A. (2009). Resveratrol pretreatment protects rat brain from cerebral ischemic damage via a sirtuin 1-uncoupling protein 2 pathway. Neuroscience 159, 993–1002. 10.1016/j.neuroscience.2009.01.017 19356683 PMC2668125

[B6] Dong Y. Fan C. Hu W. Jiang S. Ma Z. Yan X.. (2016). Melatonin attenuated early brain injury induced by subarachnoid hemorrhage via regulating NLRP3 inflammasome and apoptosis signaling. J. Pineal Res. 60, 253–262. 10.1111/jpi.12300 26639408

[B7] Fu Y. Wang Y. Du L. Xu C. Cao J. Fan T.. (2013). Resveratrol inhibits ionising irradiation-induced inflammation in MSCs by activating SIRT1 and limiting NLRP-3 inflammasome activation. Int. J. Mol. Sci. 14, 14105–14118. 10.3390/ijms140714105 23880858 PMC3742234

[B8] Guo Z. N. Xu L. Hu Q. Matei N. Yang P. Tong L. S.. (2016). Hyperbaric oxygen preconditioning attenuates hemorrhagic transformation through reactive oxygen species/thioredoxin-interacting protein/nod-like receptor protein 3 Pathway in hyperglycemic middle cerebral artery occlusion rats. Crit. Care Med. 44, e403–e411. 10.1097/CCM.0000000000001468 26646457

[B9] Koronowski K. B. Dave K. R. Saul I. Camarena V. Thompson J. W. Neumann J. T.. (2015). Resveratrol preconditioning induces a novel extended window of ischemic tolerance in the mouse brain. Stroke 46, 2293–2298. 10.1161/STROKEAHA.115.009876 26159789 PMC4519394

[B10] Lan X. Han X. Li Q. Li Q. Gao Y. Cheng T.. (2017a). Pinocembrin protects hemorrhagic brain primarily by inhibiting toll-like receptor 4 and reducing M1 phenotype microglia. Brain Behav. Immun. 61, 326–339. 10.1016/j.bbi.2016.12.012 28007523 PMC5453178

[B11] Lan X. Han X. Li Q. Yang Q. W. Wang J. (2017b). Modulators of microglial activation and polarization after intracerebral haemorrhage. Nat. Rev. Neurol. 13, 420–433. 10.1038/nrneurol.2017.69 28524175 PMC5575938

[B12] Lebeaupin C. Proics E. de Bieville C. H. Rousseau D. Bonnafous S. Patouraux S.. (2015). ER stress induces NLRP3 inflammasome activation and hepatocyte death. Cell Death Dis. 6:e1879. 10.1038/cddis.2015.248 26355342 PMC4650444

[B13] Li J. Chen J. Mo H. Chen J. Qian C. Yan F.. (2015). Minocycline protects against NLRP3 inflammasome-induced inflammation and P53-associated apoptosis in early brain injury after subarachnoid hemorrhage. Mol. Neurobiol. 53, 2668–2678. 10.1007/s12035-015-9318-8 26143258

[B14] Li Y. Yang J. Chen M. H. Wang Q. Qin M. J. Zhang T.. (2015). Ilexgenin A inhibits endoplasmic reticulum stress and ameliorates endothelial dysfunction via suppression of TXNIP/NLRP3 inflammasome activation in an AMPK dependent manner. Pharmacol. Res. 99, 101–115. 10.1016/j.phrs.2015.05.012 26054569

[B15] Lin C. J. Chen T. H. Yang L. Y. Shih C. M. (2014). Resveratrol protects astrocytes against traumatic brain injury through inhibiting apoptotic and autophagic cell death. Cell Death Dis. 5:e1147. 10.1038/cddis.2014.123 24675465 PMC3973229

[B16] Lopez M. S. Dempsey R. J. Vemuganti R. (2015). Resveratrol neuroprotection in stroke and traumatic CNS injury. Neurochem. Int. 89, 75–82. 10.1016/j.neuint.2015.08.009 26277384 PMC4587342

[B17] Ma Q. Chen S. Hu Q. Feng H. Zhang J. H. Tang J. (2014). NLRP3 inflammasome contributes to inflammation after intracerebral hemorrhage. Ann. Neurol. 75, 209–219. 10.1002/ana.24070 24273204 PMC4386653

[B18] Murphy M. P. (2009). How mitochondria produce reactive oxygen species. Biochem. J. 417, 1–13. 10.1042/BJ20081386 19061483 PMC2605959

[B19] Narayanan S. V. Dave K. R. Saul I. Perez-Pinzon M. A. (2015). Resveratrol preconditioning protects against cerebral ischemic injury via nuclear erythroid 2-related factor 2. Stroke 46, 1626–1632. 10.1161/STROKEAHA.115.008921 25908459 PMC4442036

[B20] Rege S. D. Geetha T. Griffin G. D. Broderick T. L. Babu J. R. (2014). Neuroprotective effects of resveratrol in Alzheimer disease pathology. Front. Aging Neurosci. 6:218. 10.3389/fnagi.2014.00218 25309423 PMC4161050

[B21] Sehba F. A. Hou J. Pluta R. M. Zhang J. H. (2012). The importance of early brain injury after subarachnoid hemorrhage. Prog. Neurobiol. 97, 14–37. 10.1016/j.pneurobio.2012.02.003 22414893 PMC3327829

[B22] Sehba F. A. Pluta R. M. Zhang J. H. (2011). Metamorphosis of subarachnoid hemorrhage research: from delayed vasospasm to early brain injury. Mol. Neurobiol. 43, 27–40. 10.1007/s12035-010-8155-z 21161614 PMC3023855

[B23] Shao A. Wu H. Hong Y. Tu S. Sun X. Wu Q.. (2015). Hydrogen-rich saline attenuated subarachnoid hemorrhage-induced early brain injury in rats by suppressing inflammatory response: possible involvement of NF-symbolizeB pathway and NLRP3 Inflammasome. Mol. Neurobiol. 53, 3462–3476. 10.1007/s12035-015-9242-y 26091790

[B24] Shao A. W. Wu H. J. Chen S. Ammar A. B. Zhang J. M. Hong Y. (2014). Resveratrol attenuates early brain injury after subarachnoid hemorrhage through inhibition of NF-kappaB-dependent inflammatory/MMP-9 pathway. CNS Neurosci. Ther. 20, 182–185. 10.1111/cns.12194 24279692 PMC6493158

[B25] Sui D. M. Xie Q. Yi W. J. Gupta S. Yu X. Y. Li J. B.. (2016). Resveratrol Protects against Sepsis-Associated Encephalopathy and Inhibits the NLRP3/IL-1beta Axis in Microglia. Mediat. Inflamm. 2016:1045657. 10.1155/2016/1045657 26924896 PMC4746398

[B26] Sun Q. Wu W. Hu Y. C. Li H. Zhang D. Li S.. (2014). Early release of high-mobility group box 1 (HMGB1) from neurons in experimental subarachnoid hemorrhage *in vivo* and *in vitro*. J. Neuroinflammation 11:106. 10.1186/1742-2094-11-106 24924349 PMC4107626

[B27] Wali J. A. Masters S. L. Thomas H. E. (2013). Linking metabolic abnormalities to apoptotic pathways in Beta cells in type 2 diabetes. Cells 2, 266–283. 10.3390/cells2020266 24709700 PMC3972679

[B28] Walsh J. G. Muruve D. A. Power C. (2014). Inflammasomes in the CNS. Nat. Rev. Neurosci. 15, 84–97. 10.1038/nrn3638 24399084

[B29] Wang F. Cui N. Yang L. Shi L. Li Q. Zhang G.. (2015). Resveratrol Rescues the impairments of hippocampal neurons stimulated by microglial over-activation *in vitro*. Cell Mol. Neurobiol. 35, 1003–1015. 10.1007/s10571-015-0195-5 25898934 PMC11486292

[B30] Wang Y. Kong H. Zeng X. Liu W. Wang Z. Yan X.. (2016). Activation of NLRP3 inflammasome enhances the proliferation and migration of A549 lung cancer cells. Oncol. Rep. 35, 2053–2064. 10.3892/or.2016.4569 26782741

[B31] Wang Z. Shi X. Y. Yin J. Zuo G. Zhang J. Chen G. (2012). Role of autophagy in early brain injury after experimental subarachnoid hemorrhage. J. Mol. Neurosci. 46, 192–202. 10.1007/s12031-011-9575-6 21728063

[B32] Wang Z. Wang Y. Tian X. Shen H. Dou Y. Li H.. (2016). Transient receptor potential channel 1/4 reduces subarachnoid hemorrhage-induced early brain injury in rats via calcineurin-mediated NMDAR and NFAT dephosphorylation. Sci. Rep. 6:33577. 10.1038/srep33577 27641617 PMC5027540

[B33] Wu J. Xu X. Li Y. Kou J. Huang F. Liu B.. (2014). Quercetin, luteolin and epigallocatechin gallate alleviate TXNIP and NLRP3-mediated inflammation and apoptosis with regulation of AMPK in endothelial cells. Eur. J. Pharmacol. 745, 59–68. 10.1016/j.ejphar.2014.09.046 25446924

[B34] Wu L. Y. Ye Z. N. Zhou C. H. Wang C. X. Xie G. B. Zhang X. S.. (2017). Roles of pannexin-1 channels in inflammatory response through the TLRs/NF-kappa B signaling pathway following experimental subarachnoid hemorrhage in rats. Front. Mol. Neurosci. 10:175. 10.3389/fnmol.2017.00175 28634441 PMC5459922

[B35] Wu Q. Qi L. Li H. Mao L. Yang M. Xie R.. (2017). Roflumilast reduces cerebral inflammation in a rat model of experimental subarachnoid hemorrhage. Inflammation. 40, 1245–1253. 10.1007/s10753-017-0567-8 28451841 PMC6193485

[B36] Yan H. Zhang D. Hao S. Li K. Hang C. H. (2015). Role of mitochondrial calcium uniporter in early brain injury after experimental subarachnoid hemorrhage. Mol. Neurobiol. 52, 1637–1647. 10.1007/s12035-014-8942-z 25370932

[B37] Yang F. Wang Z. Wei X. Han H. Meng X. Zhang Y.. (2014). NLRP3 deficiency ameliorates neurovascular damage in experimental ischemic stroke. J. Cereb. Blood Flow Metab. 34, 660–667. 10.1038/jcbfm.2013.242 24424382 PMC3982086

[B38] Yang S. J. Lim Y. (2014). Resveratrol ameliorates hepatic metaflammation and inhibits NLRP3 inflammasome activation. Metabolism 63, 693–701. 10.1016/j.metabol.2014.02.003 24629563

[B39] Yang X. Xu S. Qian Y. Xiao Q. (2017). Resveratrol regulates microglia M1/M2 polarization via PGC-1alpha in conditions of neuroinflammatory injury. Brain Behav. Immun. 64, 162–172. 10.1016/j.bbi.2017.03.003 28268115

[B40] You W. Wang Z. Li H. Shen H. Xu X. Jia G.. (2016). Inhibition of mammalian target of rapamycin attenuates early brain injury through modulating microglial polarization after experimental subarachnoid hemorrhage in rats. J. Neurol. Sci. 367, 224–231. 10.1016/j.jns.2016.06.021 27423593

[B41] Yuan J. Liu W. Zhu H. Zhang X. Feng Y. Chen Y.. (2017). Curcumin attenuates blood-brain barrier disruption after subarachnoid hemorrhage in mice. J. Surg. Res. 207, 85–91. 10.1016/j.jss.2016.08.090 27979493

[B42] Zhang Q. Yuan L. Zhang Q. Gao Y. Liu G. Xiu M.. (2015). Resveratrol attenuates hypoxia-induced neurotoxicity through inhibiting microglial activation. Int. Immunopharmacol. 28, 578–587. 10.1016/j.intimp.2015.07.027 26225925

[B43] Zhang T. Su J. Wang K. Zhu T. Li X. (2014). Ursolic acid reduces oxidative stress to alleviate early brain injury following experimental subarachnoid hemorrhage. Neurosci. Lett. 579, 12–17. 10.1016/j.neulet.2014.07.005 25026072

[B44] Zhang X. S. Li W. Wu Q. Wu L. Y. Ye Z. N. Liu J. P.. (2016a). Resveratrol attenuates acute inflammatory injury in experimental subarachnoid hemorrhage in rats via inhibition of TLR4 pathway. Int. J. Mol. Sci. 17:e1131. 10.3390/ijms17081331 27529233 PMC5000728

[B45] Zhang X. S. Wu Q. Wu L. Y. Ye Z. N. Jiang T. W. Li W.. (2016b). Sirtuin 1 activation protects against early brain injury after experimental subarachnoid hemorrhage in rats. Cell Death Dis. 7:e2416. 10.1038/cddis.2016.292 27735947 PMC5133967

[B46] Zhang X. S. Zhang X. Zhang Q. R. Wu Q. Li W. Jiang T. W.. (2015). Astaxanthin reduces matrix metalloproteinase-9 expression and activity inthe brain after experimental subarachnoid hemorrhage in rats. Brain Res. 1624, 113–124. 10.1016/j.brainres.2015.07.020 26210617

[B47] Zhang X. S. Zhang X. Zhou M. L. Zhou X. M. Li N. Li W.. (2014). Amelioration of oxidative stress and protection against early brain injury by astaxanthin after experimental subarachnoid hemorrhage. J. Neurosurg. 121, 42–54. 10.3171/2014.2.JNS13730 24724856

[B48] Zhang Z. Zhang Z. Lu H. Yang Q. Wu H. Wang J. (2017). Microglial polarization and inflammatory mediators after intracerebral hemorrhage. Mol. Neurobiol. 54, 1874–1886. 10.1007/s12035-016-9785-6 26894396 PMC4991954

[B49] Zhou X. Chen M. Zeng X. Yang J. Deng H. Yi L.. (2014). Resveratrol regulates mitochondrial reactive oxygen species homeostasis through Sirt3 signaling pathway in human vascular endothelial cells. Cell Death Dis. 5:e1576. 10.1038/cddis.2014.530 25522270 PMC4454164

[B50] Zhu W. Cao F. S. Feng J. Chen H. W. Wan J. R. Lu Q.. (2017). NLRP3 inflammasome activation contributes to long-term behavioral alterations in mice injected with lipopolysaccharide. Neuroscience 343, 77–84. 10.1016/j.neuroscience.2016.11.037 27923741 PMC5349320

